# High-fat diet promotes gestational diabetes mellitus through modulating gut microbiota and bile acid metabolism

**DOI:** 10.3389/fmicb.2024.1480446

**Published:** 2025-01-28

**Authors:** Lei Yao, Xuefei Zhou, Xianqi Jiang, Hao Chen, Yuanliang Li, Xiao Xiong, Yan Tang, Haogang Zhang, Pengfei Qiao

**Affiliations:** ^1^Department of General Surgery, The Second Affiliated Hospital of Harbin Medical University, Harbin, China; ^2^Department of Gynaecology and Obstetrics, The Center of Red Cross Hospital of Harbin, Harbin, China

**Keywords:** high-fat diet, gestational diabetes mellitus, gut microbiota, bile acids metabolism, Fxr pathway

## Abstract

**Introduction:**

Gestational diabetes mellitus (GDM) is a condition characterized by glucose intolerance during pregnancy, estimated to affect approximately 20% of the whole pregnancies and is increasing in prevalence globally. However, there is still a big gap in knowledge about the association between gut microbiota associated metabolism alterations and GDM development.

**Methods:**

All the participants accomplished the validated internet-based dietary questionnaire for Chinese and serum, fecal samples were collected. HFD, control diet or colesevelam intervention was fed to GDM mice models or Fxr-/- mice models, with or without antibiotics cocktail treatment. Fecal microbiota transplantation were used for further validation. Gut microbiota and metabolites were detected by metagenomic sequencing and high-performance liquid chromatography-mass spectrometry, respectively. Bile acids of serum, fecal samples from human and mice were analysised. Body weight, average feed intake, blood glucose, insulin levels and oral glucose tolerance test was performed among each groups. Expression levels of Fxr, Shp and Fgf15 mRNA and protein were detected by quantitative reverse transcription polymerase chain reaction and western blot, respectively.

**Results:**

Our data indicated that high fat diet (HFD) was linked with higher prevalence of GDM, and HFD was positively associated with poor prognosis in GDM patients. Moreover, compared with normal diet (ND) group, GDM patients from HFD group performed a loss of gut microbiota diversity and enrichment of *Alistipes onderdonkii*, *Lachnospiraceae bacterium 1_7_58FAA*, and *Clostridium aspaaragiforme* while ruduction of *Akkermansiaceae, Paraprevotell xylaniphila, and Prevotella copri*. Additionally, HFD aggravated GDM in mice and gut microbiota depletion by antibiotics crippled the effect of excess fat intake. BAs profile altered in HFD GDM patients and mice models. Fecal microbiota transplantation (FMT) further confirmed that gut microbiota contributed to bile acids (BAs) metabolic dysfunction during HFD-associated GDM development. Mechanically, HFD-FMT administration activated *Fxr, Shp,* and *Fgf15* activity, disturbed the glucose metabolism and aggravated insulin resistance but not in HFD-FMT *Fxr−/−* mice and ND-FMT *Fxr−/−* mice. Furthermore, colesevelam intervention alleviated HFD-associated GDM development, improved BAs metabolism, suppressed *Fxr, Shp,* and *Fgf15* activity only in *WT* mice but not in the *Fxr−/−* HFD + Colesevelam group and *Fxr−/−* HFD group. HFD induced GDM and contributed to poor prognosis in GDM parturients through inducing gut microbial dysbiosis and metabolic alteration, especially appeared in BAs profile. Moreover, Fxr pathway participated in regulating HFD-associated gut microbiota disordered BAs metabolites and aggravating GDM in mice.

**Discussion:**

Modulating gut microbiota and BAs metabolites could be a potential therapeutic strategy in the prevention and treatment of HFD-associated GDM.

## Introduction

Gestational diabetes mellitus (GDM) is a condition characterized by glucose intolerance during pregnancy, estimated to affect approximately 20% of the whole pregnancies and is increasing in prevalence globally (Hannah et al., 2023). GDM is associated with obstetric and neonatal complications primarily due to increased birthweight and is a major risk factor for future type 2 diabetes, obesity, and cardiovascular disease in mother and offspring (Greco et al., 2024). As one of the most important factors, excess dietary fat intake is intensively associated with increased GDM risk (Cole et al., 2021a). However, there is still a big gap in knowledge, and more investigation is needed to determine the association between dietary fat intake and GDM development.

As we known, the composition of gut microbiota affects the occurrence and development of GMD (Ponzo et al., 2019). Furthermore, microbe-derived metabolites, such as bile acids, short-chain fatty acids, branched-chain amino acids, trimethylamine N-oxide, tryptophan, and indole derivatives, have been implicated in the pathogenesis of metabolic disorders (Vladu et al., 2022). Established viewpoints reminded that perturbations in the gut microbiome could affect the BAs pool size and composition, which were linked to metabolic disorders, including GDM development (Maghsoodi et al., 2019). Both excess dietary fat intake and imbalanced gut microbiome are correlated with GDM; however, the intricate relationship between high-fat diet (HFD) and microbiome dysbiosis and its metabolites changes in GDM development is largely unknown.

In this study, we demonstrated HFD-derived GDM progression through inducing gut microbial dysbiosis with enrichment of pathogenic bacteria and depletion of probiotic, along with bile acids metabolomic dysregulation, confirming that gut microbiota plays an important role in HFD-induced GDM development.

## Materials and methods

### Patients and the blood, fecal samples

We used data from the validated Internet-based dietary questionnaire for Chinese^1^ (Feng et al., 2016). Our study included 50 healthy puerperae and 50 GDM patients enrolled from March 2020 to March 2024 in northern Chinese area. The exclusive criteria were as follows: (1) smoking; (2) extreme daily energy intake (<600 kcal/day or >4,000 kcal/day); (3) alcohol drinking; (4) diagnosed of diabetes before pregnancy; (5) received antibiotic or probiotics treatment within 6 weeks; (6) received insulin therapy within 6 weeks. All participants provided written informed consent according to our institutional guidelines, and the study protocol was approved by the Institutional Review Board of Harbin Medical University (KY2019-068). Data of clinical characteristics were collected from medical records. Serum and fecal samples were collected and stored at −80°C until analysis.

### Animal models and experimental protocols

Six-week-old C57BL/6J mice were obtained from Animal Center of the Second Affiliated Hospital of Harbin Medical University. Six-week-old C57BL/6J *Fxr−/−* and *WT* mice were obtained from Vitalstar Biotechnology Co., Ltd. (Beijing, China). Mice were acclimated in fresh food and water ad libitum, temperature (22.0°C ± 1°C), humidity (40–60%), and light (12-h/12-h light/dark). Experiments were approved by the Institutional Review Board of Harbin Medical University (SYDW2019-93). Vaginal smears were performed and examined under a microscope to confirm mating successfully, at a 2:1 ratio of females to males. All mice were intraperitoneally injected with 30 mg/kg STZ (dissolved in 0.1 M sodium citrate buffer, Solarbio, Beijing, China) on Day 0 of pregnancy (D0). Blood glucose levels were monitored using a blood glucose meter after 3 days of feeding. Mice with fasted blood glucose levels >11.1 mM were considered GDM models (Zhou et al., 2020). Mice with GDM were arbitrarily assigned to 1 of 3 groups: ND, HFD, or HFD + ABX, in a generalized randomized complete block design, with 10 mice per group. Mice in ND group were fed with control rodent diet (18% protein, 5% fat), and mice in HFD group were fed with HFD (20% protein, 60% total fat, 32.1% saturated fat). HFD + ABX group mice were given drinking water with an antibiotics cocktail (0.2 g/L ampicillin, neomycin, and metronidazole and 0.1 g/L vancomycin) to deplete the gut microbiota for 2 weeks before the experiment and every other 2 weeks throughout the entire experiment.

In the fecal microbiota transplantation (FMT) study, the microbiota donors were mice fed with ND or HFD for 8 weeks. Feces of the donors were collected and dispersed in sterile Ringer working buffer, and the supernatant was prepared for transplantation. The GDM mice were randomly divided into two groups (10 each group), housed in sterile plastic package isolators (each group for one isolator), and supplied with sterilized normal diet. After 1-week acclimation, mice were oral gavaged with fecal suspension from mice with ND (ND-FMT) or HFD (HFD-FMT). The oral gavage was repeated in the next 2 days to reinforce the microbiota transplantation. All the mice were given drinking water with an antibiotics cocktail to deplete the gut microbiota for 2 weeks before the experiment and every other 2 weeks throughout the entire experiment. The body weight and average food intake were recorded. Blood and fecal samples were collected and stored at −80°C until analysis. After the mice were euthanized, the distal ileum (lower third of the small intestine), liver, and colon were collected. The tissues were directly transferred into sterile EP tubes. All these procedures were conducted in an ice bath.

For the FXR regulation study, we used colesevelam, a BAs sequestrant, which could bind BAs in the intestine, lead to the formation of non-absorbable complexes that were excreted into the feces, and thereby promoted fecal BA excretion. *Fxr−/−* and *WT* mice were fed with a standard diet supplemented or not with 2% of colesevelam hydrochloride (Daiichi Sankyo Pharma Development, Edison, NJ, USA) for a period of 8-week colesevelam intervention. Then, mice from the *WT* HFD + Colesevelam and *Fxr−/−* HFD + Colesevelam groups were subsequently treated with colesevelam mixed into the HFD, while the other animals (*WT* HFD group and *Fxr−/−* HFD group) remained on HFD without colesevelam treatment. Moreover, in the cholic acid feeding study, *Fxr−/−* and *WT* mice were fed with a high-fat diet supplemented or not with 1% cholic acid oral gavaged (CA, Chengdu MUST BIO-Technology CO., LTD, 100 mg/kg) for 8 weeks.

### Metagenome DNA extraction and shotgun sequencing

Total microbial genomic DNA samples were extracted using the OMEGA Mag-Bind Soil DNA Kit (M5635-02) (Omega Bio-Tek, Norcross, GA, USA), following the manufacturer’s instructions, and stored at −20°C prior to further assessment. The quantity and quality of extracted DNAs were measured using a Qubit™ 4 Fluorometer, with WiFi: Q33238 (Qubit™ Assay Tubes: Q32856; Qubit™ 1X dsDNA HS Assay Kit: Q33231) (Invitrogen, USA) and agarose gel electrophoresis, respectively. The extracted microbial DNA was processed to construct metagenome shotgun sequencing libraries with insert sizes of 400 bp by using Illumina TruSeq Nano DNA LT Library Preparation Kit. Each library was sequenced by Illumina NovaSeq platform (Illumina, USA) with PE150 strategy at Personal Biotechnology Co., Ltd. (Shanghai, China).

### Metagenomics analysis

Raw sequencing reads were processed to obtain quality-filtered reads for further analysis. First, sequencing adapters were removed from sequencing reads using Cutadapt (v1.2.1). Second, low-quality reads were trimmed using a sliding-window algorithm in fastp. Third, reads were aligned to the host genome using BMTagger to remove host contamination. Once quality-filtered reads were obtained, taxonomical classifications of metagenomics sequencing reads from each sample were performed using Kraken2 against an RefSeq-derived database, which included genomes from archaea, bacteria, viruses, fungi, protozoans, metazoans, and viridiplantae. Reads assigned to metazoans or viridiplantae were removed for downstream analysis. Megahit (v1.1.2) was used to assemble for each sample using the meta-large presetted parameters. The generated contigs (longer than 300 bp) were then pooled together and clustered using mmseqs2 with “easy-linclust” mode, setting sequence identity threshold to 0.95 and covered residues of the shorter contig to 90%. The lowest common ancestor taxonomy of the non-redundant contigs was obtained by aligning them against the NCBI-nt database by mmseqs2 with “taxonomy” mode, and contigs assigned to Viridiplantae or Metazoa were dropped in the following analysis. MetaGeneMark was used to predict the genes in the contigs. CDS sequences of all samples were clustered by mmseqs2 with “easy-cluster” mode, setting protein sequence identity threshold to 0.90 and covered residues of the shorter contig to 90%. To assess the abundances of these genes, the high-quality reads from each sample were mapped onto the predicted gene sequences using salmon in the quasi-mapping-based mode with “--meta --minScoreFraction = 0.55,” and the CPM (copy per kilobase per million mapped reads) was used to normalize abundance values in metagenomes. The functionality of the non-redundant genes was obtained by annotated using mmseqs2 with the “search” mode against the protein databases of KEGG, EggNOG, and CAZy databases, respectively. EggNOG and GO were obtained using EggNOG-mapper (v2). GO ontology was obtained using map2slim.^2^ KO were obtained using KOBAS.

### Untargeted metabolomics data preprocessing and analysis

Stool samples were quickly frozen in liquid nitrogen after collection and stored at −80°C until analysis. The LC analysis was performed on a Vanquish UHPLC System (Thermo Fisher Scientific, USA). Chromatography was carried out with an ACQUITY UPLC® HSS T3 (2.1 × 100 mm, 1.8 μm) (Waters, Milford, MA, USA). The column was maintained at 40°C. The flow rate and injection volume were set at 0.3 mL/min and 2 μL, respectively. For LC-ESI (+)-MS analysis, the mobile phases consisted of (B1) 0.1% formic acid in acetonitrile (v/v) and (A1) 0.1% formic acid in water (v/v). Separation was conducted under the following gradient: 0–1 min, 8% B1; 1–8 min, 8–98% B1; 8–10 min, 98% B1; 10–10.1 min, 98–8% B1; 10.1–12 min, 8% B1. For LC-ESI (−)-MS analysis, the analytes was carried out with (B2) acetonitrile and (A2) ammonium formate (5 mM). Separation was conducted under the following gradient: 0–1 min, 8% B2; 1–8 min, 8–98% B2; 8–10 min, 98% B2; 10–10.1 min, 98–8% B2; 10.1–12 min, 8% B2.

### BAs analysis

Each 10 mg fecal sample or 5 μL serum content sample was extracted by a two-step extraction. A 200-μL aliquot of methanol/water (1:1, containing the six internal standards CA-d4, LCAd4, UDCA-d4, GCA-d4, GCDCA-d4, and glycodeoxycholic acid-d4, at 50 nM each) was added to the sample. The sample was homogenized for 5 min and centrifuged at 13,200 g at 4°C for 15 min. The supernatant was transferred into a 1.5 mL tube, and the sample residue was further extracted by a 200 μL aliquot of methanol/acetonitrile (2:8, containing the six internal standards as the first extraction solvent). After homogenization and centrifugation, the supernatant was transferred to the tube with the first extraction. The extraction mixture was vortexed for 3 min and centrifuged at 13,200 *g* at 4°C for 15 min. The supernatant was then transferred to a sampling vial for analysis. BA analysis was performed on the instrument UPLC/TQMS. The elution solvents were water +0.01% formic acid (A) and acetonitrile/methanol (19:1) + 0.01% formic acid (B). The elution gradient over 20 min at a flow rate of 450 μL/min was as follows: 0–2 min (20% B), 2–3 min (20–25% B), 3–6 min (25% B), 6–8 min (25–35% B), 8–11.5 min (35% B), 11.5–18 min (35–99% B), 18–19 min (99% B), and 19–20 min (99–20% B). The MS was operated at a negative electrospray ionization mode. The cone and collision energy for each BA used the optimized settings from QuanOptimize application manager (Waters Corp., Milford, MA, USA). One standard calibration solution at 10 different concentration levels contains 45 standards and was tested every 40 samples. The peak annotation and quantitation was performed by TargetLynx Application Manager.

### Body weight, average feed intake, blood glucose, and insulin analysis

The body weight of mice was recorded before dietary intervention at D0, D6, D7, D12, D13, and D18. Then, average feed intake was recorded at the same time point. Body weight was recorded on a top-loading balance (Fisher Scientific, Suwanee, GA, USA). Non-fasting maternal blood samples were obtained via tail venipuncture to detect blood glucose and plasma insulin levels at D0, D6, D12, and D18. Blood glucose levels were determined by glucometer (LifeScan SureStep; Johnson & Johnson, Langhorne, PA, USA), and plasma insulin levels were quantified by enzyme-linked immunosorbent assay (ELISA; Alpco Diagnostics, Salem, NH, USA) according to the manufacturer’s instructions.

### Oral glucose tolerance test (OGTT)

Fast the mice for 6 h before OGTT. Then, the glucose bolus was delivered intragastrically using a feeding needle. Routinely, blood glucose concentrations were measured at 0, 0.5, 1.0, 1.5, and 2.0 h after glucose delivery, and the results were recorded in the data sheet.

### Western blot analysis

Total protein was extracted from tissues, and the protein concentration was measured by BCA Protein Assay Kit (Beyotime Institute of Biotechnology, China). The primary antibodies, including Fxr, Shp, Fgf15, and β-actin, were purchased from Santa Cruz Biotechnology (USA). Enhanced chemiluminescence detection reagents of donkey anti-mouse IgG Alexa Fluor 680 or donkey anti-rabbit IgG Alexa Fluor 680 (Thermo Fisher Scientific, USA) were incubated with the membranes for 12 h and visualized using an Odyssey Infrared Imaging system (LI-COR Biosciences, USA). Densitometry was performed using Alpha Imager 2200.

### Real-time quantitative reverse transcription PCR (RT-qPCR)

Total RNA of tissue samples was isolated by TRIzol reagent (Invitrogen, USA). Then, cDNA was synthesized by High Capacity cDNAs Reverse Transcription kit (Thermo Fisher Scientific, USA). The mRNA expression levels of target genes were evaluated by RT-qPCR with the Power®SYBR Green (Thermo Fisher Scientific, USA) through the ABI Step One system. The level of β-actin was used as internal control. The 2^−∆∆Ct^ method was used to determine the relative expression levels of the target genes. Primers used in this study are listed in Supplementary Table 1.

### Statistical analysis

SPSS 23.0 software (IBM, Chicago, IL, USA) was used to analyze the data, and the normality and homogeneity of variance of the data were tested before further analysis. Generally, for comparisons of more than two groups, an analysis of variance (ANOVA) was used. Differences between two groups were performed with *t-test* analysis. When the data were skewed or the variance was not uniform, non-parametric tests were used to analyze significant differences between groups. Comparisons of categorical variables between two groups were performed using the chi-square test or Fisher’s exact test. Based on the taxonomic and functional profiles of non-redundant genes, linear discriminant analysis effect size (LEfSe) was performed to detect differentially abundant taxa and functions across groups using the default parameters. Beta diversity analysis was performed to investigate the compositional and functional variation of microbial communities across samples using Bray–Curtis distance metrics and visualized via principal coordinate analysis (PCoA), nonmetric multidimensional scaling (NMDS), and unweighted pair-group method with arithmetic means (UPGMA) hierarchical clustering. Graphing was performed in Graph Pad Prism 8.0 software (La Jolla, CA, USA). All the data were shown as the mean ± SD. A *p*-value of < 0.05 was considered statistically significant.

## Results

### HFD induced GDM and poor prognosis clinically

First of all, according to the diary food intake data selected from the validated Internet-based dietary questionnaire for Chinese, no significant differences in age and body mass index (BMI) were observed between the two groups. With adjustment for the above factors, HFD was linked with higher prevalence of GDM [OR 95% C.I. of 0.343 (0.152–0.775), *p* = 0.010]. Then, the 50 GDM parturients were divided into two groups according to HFD or not to evaluate the association between HFD and clinicopathological characteristics of GDM parturients (Table 1). Our data indicated that HFD was positively associated with high prevalence of preeclampsia, gestational hypertension, hydramnios, urinary tract or vaginal infections, and cesarean delivery. However, age, BMI, perineal tears, postpartum hemorrhage, difficulty initiating, and maintaining breastfeeding had no association with HFD. These results suggested that HFD might induce GDM and contribute to poor prognosis in GDM parturients.

**Table 1:** Association between HFD and clinicopathological characteristics of GDM parturients.

Characteristics	*N*	Diet habit		*p-*value
		ND	HFD	
Age				0.148
<35 years	25	13	12	
≥35 years	25	7	18	
BMI				0.353
<30 kg/m^2^	14	4	10	
≥30 kg/m^2^	36	16	20	
High prevalence of preeclampsia				0.041
Present	19	4	15	
Absent	31	16	15	
Gestational hypertension				0.002
Present	18	2	16	
Absent	32	18	14	
Hydramnios				0.008
Present	19	3	16	
Absent	31	17	14	
Urinary tract or vaginal infections				0.026
Present	30	18	12	
Absent	20	2	18	
Cesarean delivery				0.016
Present	12	1	11	
Absent	38	19	19	
Perineal tears				1.000
Present	12	5	7	
Absent	38	15	23	
Postpartum hemorrhage				0.672
Present	6	3	3	
Absent	44	17	27	
Difficulty initiating				1.000
Present	9	4	5	
Absent	41	16	25	
Maintaining breastfeeding				0.773
Present	22	8	14	
Absent	28	12	16	

*p < 0.05 is significant.*

### Gut microbiota alterations in GDM parturients with HFD

**Figure 1:**
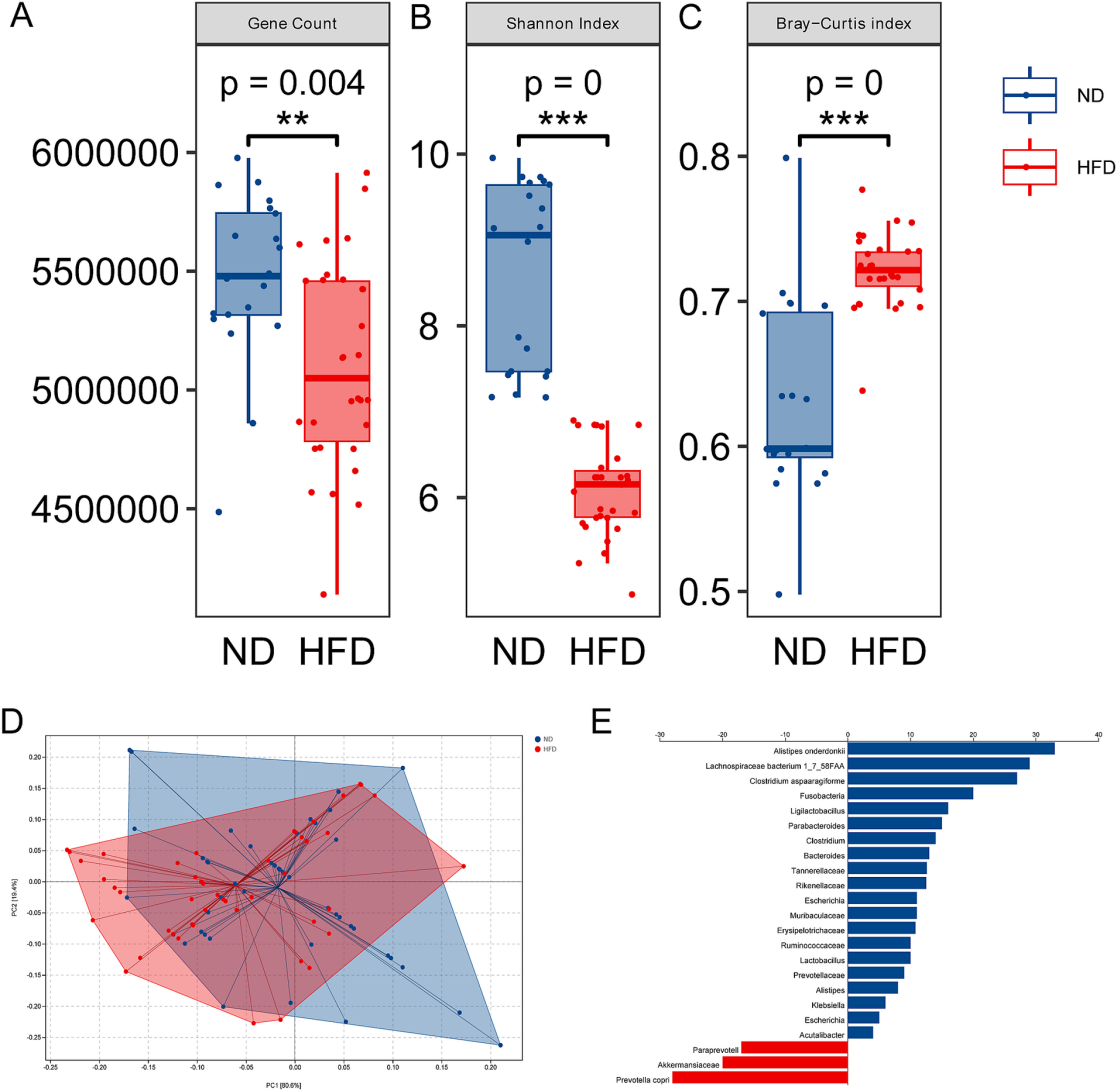
Alterations of gut microbiota in GDM with HFD. **(A)** Gene count in the ND and HFD groups. **(B)** Shannon’s index in the ND and HFD groups. **(C)** The β-diversity in the HFD group compared to the ND group. **(D)** Principal coordinate analysis of Bray–Curtis distance based on species-level between the ND and HFD groups; **(E)** LEfSe scores computed for differentially abundant species in the ND and HFD group. ***p < 0.001.

The above newly diagnosed parturients with GDM were divided into two groups according to HFD or not to evaluate gut microbiota alteration associated with excess fat intake. Fecal samples were collected and analyzed by metagenomic sequencing. Indicators of α-diversity, both gene count and Shannon’s index, were significantly decreased in the HFD group, as compared with the ND group (Figure 1A, *p* = 0.004, and Figure 1B, *p* = 0.000), suggesting a loss of gut microbiota diversity in GDM patients with HFD. Revealed by Bray–Curtis index, the significant increase of heterogeneity in gut microbiota was found in HFD groups (Figure 1C, *p* = 0.000). Then, the dramatic differences were also found in β-diversity indicated distinct microbiome profile in GDM patients between the ND and HFD groups (Figure 1D). These findings implied the possible role of gut microbiota in the development of GDM with HFD. To search for the candidate species which contributed to GDM, we proceeded to linear discriminant analysis and identified several differential species (Figure 1E). Interestingly, GDM patients with HFD were more likely attributed to decreased symbiotic microbes and increased pathogenic microbes. Among these differential species, 33 were negatively correlated with GDM with HFD, while only 10 species were enriched in the GDM patients with HFD group. Notably, 3 out of 33 enrichment of species were found from HFD group, namely, *Alistipes onderdonkii*, *Lachnospiraceae bacterium 1_7_58FAA*, and *Clostridium aspaaragiforme*. Meanwhile, *Akkermansiaceae, Paraprevotell xylaniphila, and Prevotella copri* were reduced in the HFD group. The above results suggested that HFD could promote GDM development at least partly through regulating gut microbiota alterations.

### Gut microbiota associated metabolites alterations in GDM parturients with HFD

**Figure 2:**
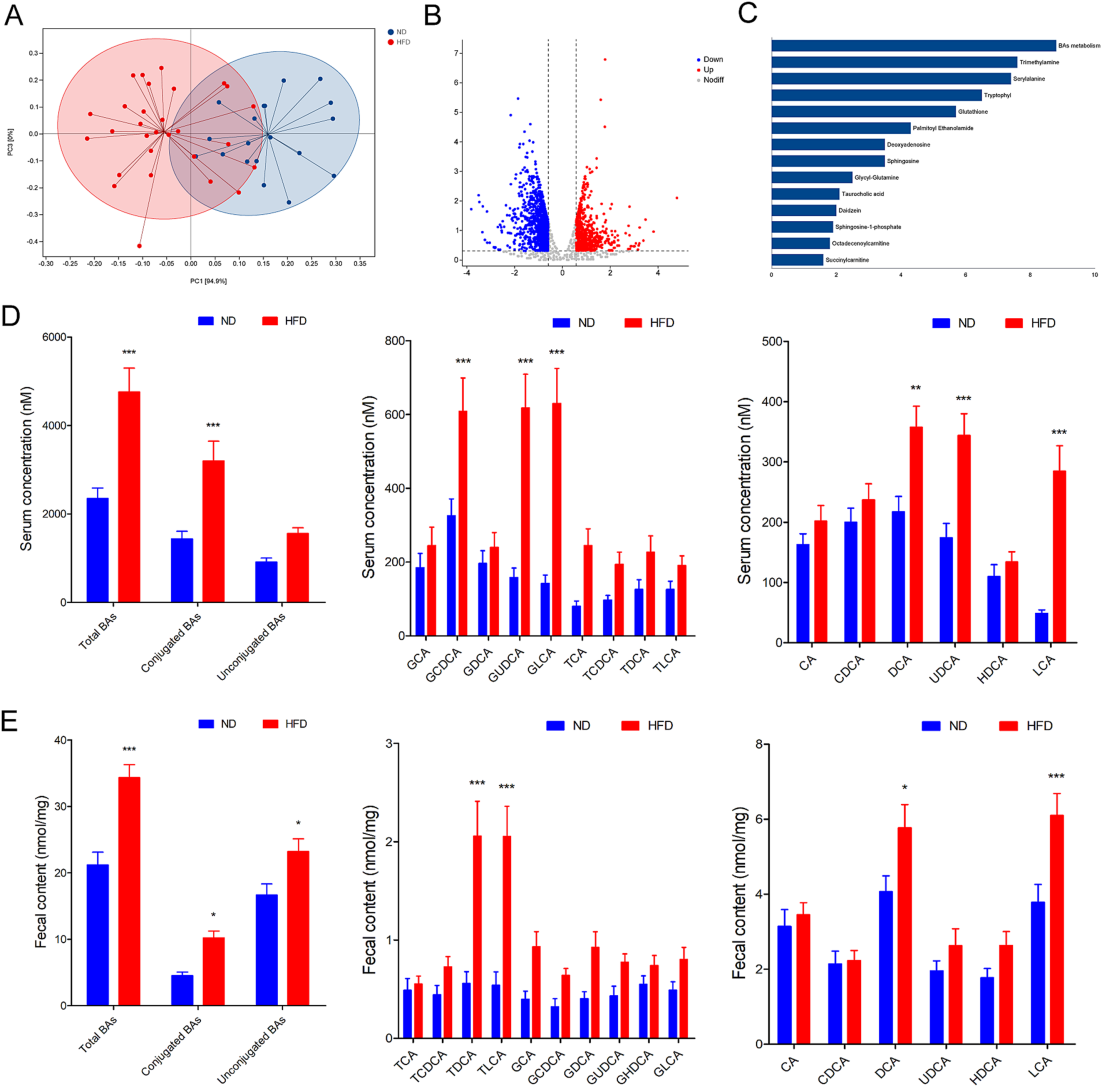
Gut microbiota associated metabolite alterations in GDM parturients with HFD. **(A)** The fecal metabolic profile alterations among each groups according to unsupervised PCA analysis. **(B)** Volcano plot for differential metabolites in comparison between ND and HFD group in GDM parturients. **(C)** Pathway analysis of differentially enriched metabolites in HFD GDM parturients compared with ND GDM parturients. **(D)** Serum BA classes and profile in GDM parturients. **(E)** Fecal BA classes and profile in GDM parturients. *p < 0.05; **p < 0.01; ***p < 0.001.

Considering the regulatory roles of the microbiota in metabolites, we performed metabolic profiling of the fecal samples by LC–MS to examine metabolic changes in GDM patients with HFD. According to unsupervised PCA analysis, the fecal metabolic profiles significantly differed among each groups (Figure 2A). Differential fecal metabolites were also identified between each group and were found to be enriched in different metabolomic signaling pathways (Figure 2B). Among these pathways, bile acids (BAs) metabolism, trimethylamine, and serylalanine were the top three significantly altered pathway in GDM patients with HFD (Figure 2C). Then, the ultra-performance liquid chromatography triple-quadrupole mass spectrometry (UPLC/TQMS)-based targeted metabolomics approach was used to analyze the levels of BAs in the serum and feces of GDM patients. The results revealed that the serum levels of the conjugated BAs, unconjugated Bas, and total BAs were significantly increased in HFD group compared with the ND group. Among them, unconjugated BAs, UDCA, DCA, and LCA performed significantly increased levels, while conjugated BAs, glyco-chenodeoxycholic acid (GCDCA), GLCA, and glycol-ursodeoxycholic acid (GUDCA) were significantly increased in human serum (Figure 2D). Furthermore, the levels of total BAs, conjugated Bas, and unconjugated BAs in fecal excretion were significantly increased in the HFD group compared with the ND group. Among them, unconjugated BAs, DCA, and LCA performed significantly increased levels, while conjugated BAs, TLCA, and TDCA were markedly increased in human fecal excretion (Figure 2E). These results suggested that BAs profile altered might be involved in modulating HFD inducing GDM development regulated by gut microbiota.

### HFD promoted GDM development dependent on gut microbiota in STZ-induced mouse models

**Figure 3:**
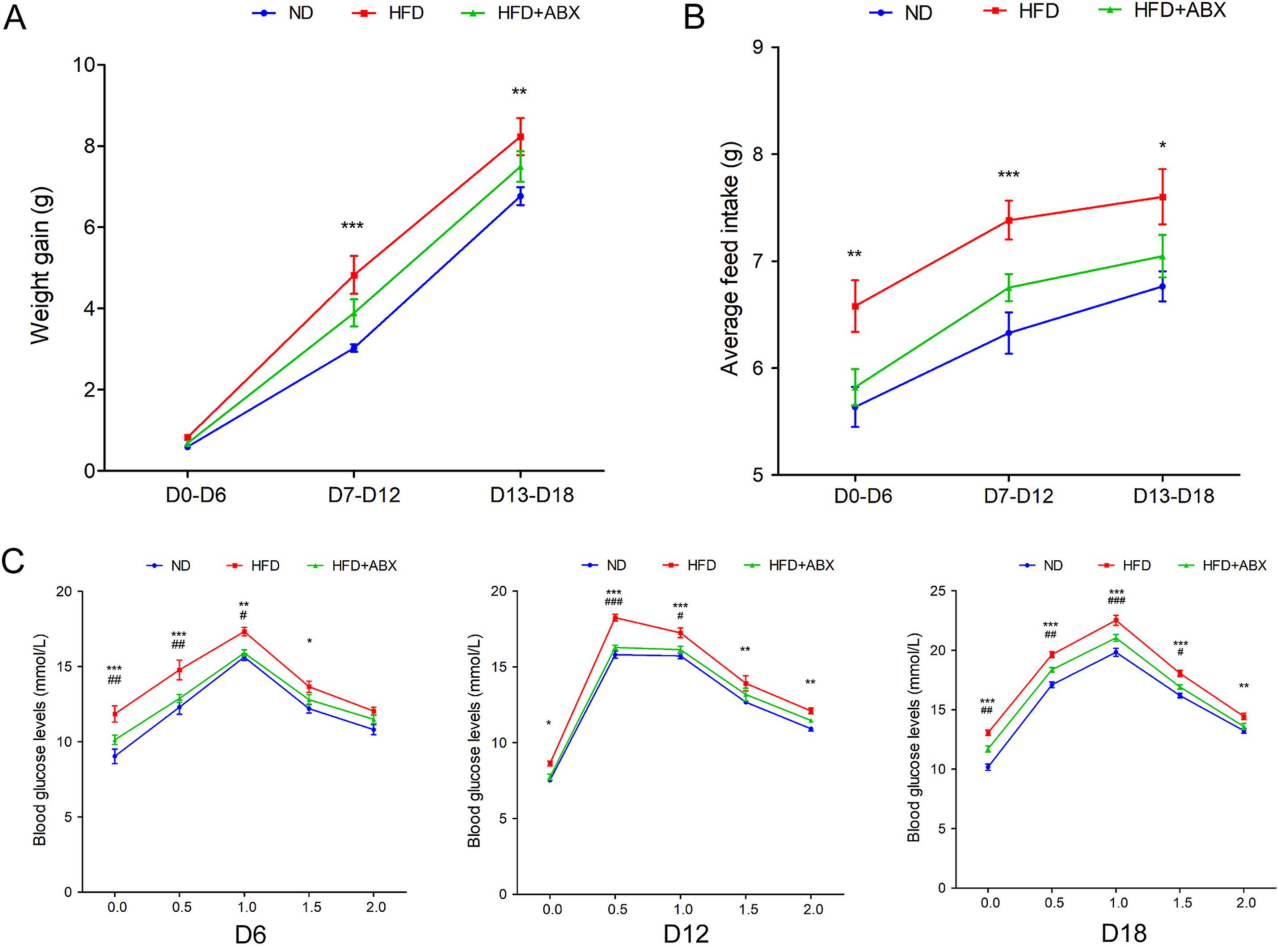
HFD promoted GDM development dependent on gut microbiota in STZ-induced mouse models. **(A)** The line chart showed the body weight gain among each groups during different pregnancy periods. **(B)** The barplot showed average feed intake among each groups during different pregnancy periods. **(C)** The line chart showed the results of the OGTT during different pregnancy periods. *p < 0.05; **p < 0.01; ***p < 0.001 means a significant difference versus the ND group; ^#^*p* < 0.05; ^##^*p* < 0.01; ^###^*p* < 0.001 means a significant difference versus the HFD group.

To examine the role of HFD in GDM, we fed GDM mice with either HFD or ND, along with or without periodic antibiotics cocktail treatment. Mice body weight was significantly increased in pregnancy (D7–D12 and D13–D18) in the HFD group compared to the ND group, while antibiotic treatment impaired the enhanced effect on body weight of HFD-fed mice (Figure 3A). Then, the average feed intake of mice in pregnancy (D0–D6, D7–D12, and D13–D18) was compared, and the food intake of HFD mice was found to be significantly more than that of the ND and HFD + ABX groups, whereas gut microbiota depletion by antibiotics impaired the enhanced effect on feed intake of HFD-fed mice (Figure 3B). To study glucose metabolism during pregnancy, the OGTT test was performed on mice 6 days of pregnancy (D6), 12 days of pregnancy (D12), and 18 days of pregnancy (D18). The blood glucose levels of the mice in the HFD group were higher than those of the ND and HFD + ABX groups. However, gut microbiota depletion by antibiotics crippled the effect of excess fat intake on glucose metabolism in GDM mice (Figure 3C). Moreover, FPG, FINS, and HOMA-IR were measured at the same time points, and we found FPG, FINS, and HOMA-IR continue to increase throughout pregnancy in all of the experimental groups except for FPG on D0 and FINS on D18, and mice from HFD + ABX group showed significantly lower during this period compared with the ND group (Table 2).

**Table 2:** FPG, FINS, and HOMA-IR were measured in mice from the ND, HFD, and HFD + ABX groups.

	FPG (mmol/L)				FINS (μg/L)				HOMA-IR			
	D0	D6	D12	D18	D0	D6	D12	D18	D0	D6	D12	D18
ND	7.010 ± 1.579	6.160 ± 0.550	7.190 ± 0.580	10.000 ± 0.852	0.792 ± 0.107	0.835 ± 0.118	0.990 ± 0.185	1.070 ± 0.184	0.245 ± 0.057	0.229 ± 0.037	0.287 ± 0.052	0.476 ± 0.094
HFD	7.640 ± 1.190	7.910 ± 1.639	8.630 ± 0.508	12.160 ± 1.452	1.262 ± 0.133	1.246 ± 0.264	1.429 ± 0.397	1.194 ± 0.226	0.430 ± 0.092	0.437 ± 0.131	0.548 ± 0.157	0.650 ± 0.159
HFD + ABX	7.670 ± 1.434	6.600 ± 0.639	7.490 ± 0.605	10.670 ± 1.019	1.010 ± 0.103	1.014 ± 0.118	1.119 ± 0.205	1.119 ± 0.205	0.280 ± 0.079	0.262 ± 0.0.49	0.313 ± 0.047	0.507 ± 0.080
ND vs HFD *t* value	1.52	4.221	3.373	5.21	5.204	4.55	5.912	1.373	4.282	4.832	6.037	4.036
ND vs HFD *p-*value	*p* > 0.05	*p* < 0.001	*p* < 0.01	*p* < 0.001	*p* < 0.001	*p* < 0.001	*p* < 0.001	*p* > 0.05	*p* < 0.001	*p* < 0.001	*p* < 0.001	*p* < 0.001
HFD vs HFD + ABX *t* value	0.072	3.16	2.75	3.594	4.916	3.919	5.414	1.428	3.467	4.052	5.442	3.32
HFD vs HFD + ABX *p-*value	*p* > 0.05	*p* < 0.01	*p* < 0.05	*p* < 0.01	*p* < 0.001	*p* < 0.001	*p* < 0.001	*p* > 0.05	*p* < 0.01	*p* < 0.001	*p* < 0.001	*p* < 0.01

*p < 0.05 is significant.*

### Gut microbiota dysbiosis associated with HFD induced BAs metabolic disorders in mice

**Figure 4:**
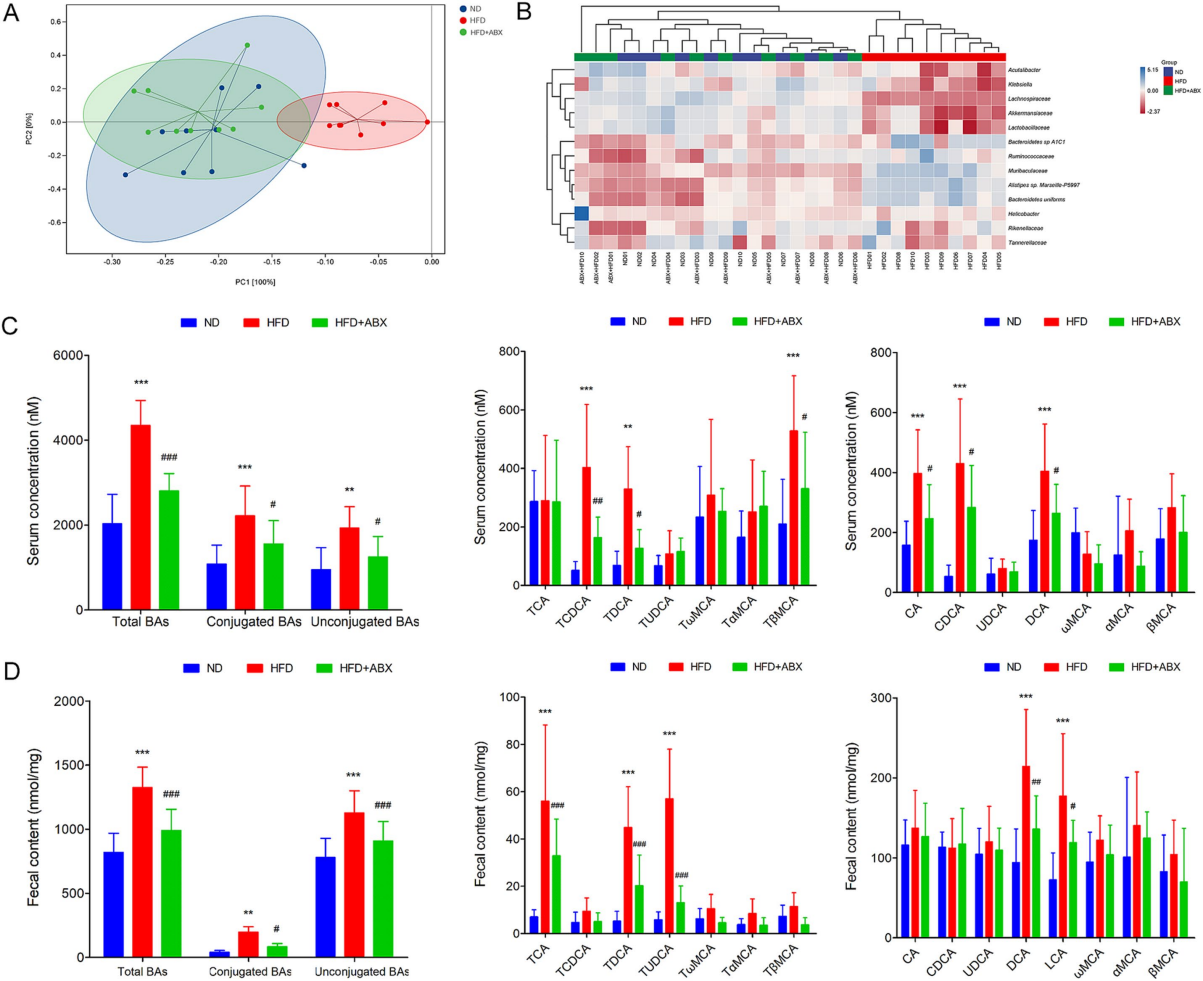
Gut microbiota dysbiosis associated with HFD induced BAs metabolic disorders in mice. **(A)** Principal component analysis showed microbiota compositional discriminations among each group. **(B)** The abundances of bacterial taxa were shown among each group. **(C)** Serum BA classes and profile in mice from each group. **(D)** Fecal BA classes and profile in mice from each group. **p < 0.01; ***p < 0.001 means a significant difference versus the ND group; ^#^*p* < 0.05; ^##^*p* < 0.01; ^###^*p* < 0.001 means a significant difference versus the HFD group.

To explore the potential involvement of gut microbial dysbiosis in HFD-associated GDM development, we performed shotgun metagenomic sequencing on stool samples of mice from each group. Microbiota compositional discriminations were observed among each group using principal component analysis (PCA). Lower bacterial diversity and reduced bacterial richness were observed in HFD mice compared with ND mice. Interestingly, there was no significantly difference in bacterial diversity and bacterial richness between the ND and HFD + ABX groups. However, compared with the HFD group, bacterial diversity and bacterial richness were markedly increased in mice from the HFD + ABX group (Figure 4A). Moreover, several bacterial taxa were differentially present in mice fed with HFD. The abundances of potential pathogenic bacterial species including *Alistipes* sp. *Marseille-P5997*, *Bacteroidetes uniforms, and Bacteroidetes* sp. *A1C1* were significantly higher in mice from the HFD group than that of mice from the ND group; whereas three protective bacterial species, namely, *Lachnospiraceae, Akkermansiaceae, and Lactobacillaceae,* were significantly depleted in mice from HFD group. Importantly, compared with the ND group, the HFD + ABX group performed no obviously difference in the abundances of bacterial species in mice. Compared with HFD group, the abundances of potential pathogenic were decreased and protective bacterial species were increased in mice from the HFD + ABX group (Figure 4B). In keeping with data clinically, the serum levels of the conjugated BAs, unconjugated Bas, and total BAs were significantly increased in the HFD group compared with the ND group. Among them, unconjugated BAs, CA, CDCA, and DCA performed significantly increased levels, while conjugated BAs, TCDCA, and TDCA were significantly increased (Figure 4C). Meanwhile, the levels of total BAs, conjugated Bas, and unconjugated BAs in fecal excretion were significantly increased in the HFD group compared with the ND group. Among them, unconjugated BAs, DCA, and LCA performed significantly increased levels, while conjugated BAs, TCA, TUDCA, and TDCA were significantly increased in the HFD group compared with the ND group (Figure 4D). On the other hand, bacteria depletion in HFD-fed mice by antibiotics partially improved BAs metabolic, as shown by decreased levels of total BAs, conjugated Bas, and unconjugated BAs in the serum (Figure 4C) as well as the fecal BA excretion (Figure 4D) compared to the HFD group. Taken together, these results indicated that HFD disturbed BAs metabolic disorders contributed to GDM development at least partly induced by gut microbial dysbiosis.

### HFD-associated gut microbiota directly confused BAs metabolic and promoted GDM development in mice

**Figure 5:**
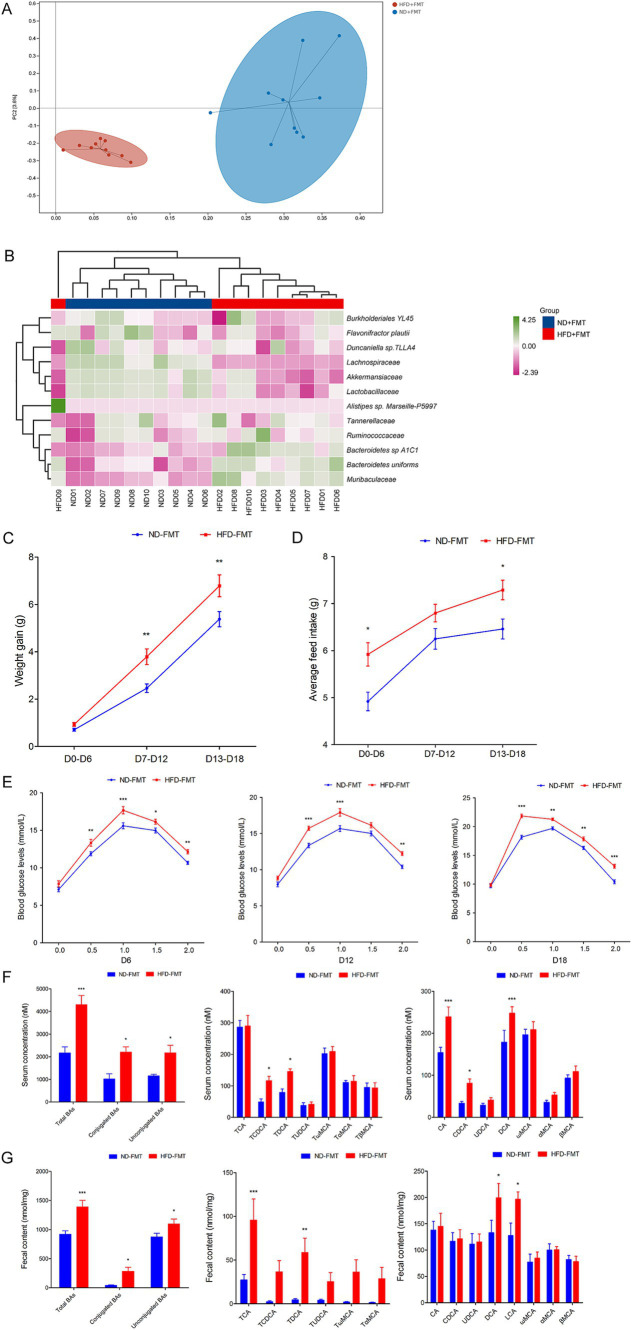
FMT affected the structure of the gut microbiota, promoted GDM development, and confused BAs metabolic in mice. **(A)** Principal component analysis showed microbiota compositional discriminations between the ND-FMT and HFD-FMT groups. **(B)** The abundances of bacterial taxa were shown between the ND-FMT and HFD-FMT groups; **(C)** The line chart showed the body weight gain between the ND-FMT and HFD-FMT groups during different pregnancy periods. **(D)** The barplot showed average feed intake between the ND-FMT and HFD-FMT groups during different pregnancy periods. **(E)** The line chart shows the results of the OGTT between the ND-FMT and HFD-FMT groups during different pregnancy periods. **(F)** Serum BA classes and profile in mice between the ND-FMT and HFD-FMT groups; **(G)** Fecal BA classes and profile in mice between the ND-FMT and HFD-FMT groups. *p < 0.05; **p < 0.01; ***p < 0.001.

To evaluate the direct effect of HFD-modulated gut microbiota on BAs metabolic and GDM development, GDM mice were gavaged with stools from ND-fed mice (ND-FMT) or HFD-fed mice (HFD-FMT), along with periodic antibiotics cocktail treatment. We initially observed lower bacterial diversity and reduced bacterial richness in HFD-FMT mice compared with ND-FMT mice (Figure 5A). Then, we evaluated the composition of gut microbiota in recipient mice and found that gut microbiota was significantly different as evidenced by the enriched potential pathogenic *Alistipes* sp. *Marseille-P5997, Bacteroidetes uniforms, and Bacteroidetes* sp. *A1C1* and depletion of *Lachnospiraceae, Akkermansiaceae, and Lactobacillaceae* in HFD-FMT mice compared with ND-FMT mice (Figure 5B). HFD-FMT mice in pregnancy (D7–D12 and D13–D18) performed markedly increased in body weight compared with ND-FMT mice (Figure 5C). Moreover, tools from HFD-fed mice significantly increased the average feed intake of mice in pregnancy (D0–D6 and D13–D18) (Figure 5D). In addition, the blood glucose levels of the mice in the HFD-FMT group were higher than those of the ND-FMT group (Figure 5E). As shown in Table 3, FPG on D6, D12, and D18 and FINS on D0, D6, D12, and HOMA-IR throughout pregnancy were significantly elevated in the HFD-FMT group compared with ND-FMT mice. To mechanically understand HFD-associated gut microbiota promote GDM development through confusing BAs metabolic, we demonstrated the levels of total BAs, conjugated Bas, and unconjugated BAs in serum were dramatically increased, specifically performed as increased levels of CA, CDCA, and DCA, along with conjugated BAs, TCDCA, and TDCA in mice from the HFD-FMT group than that of mice from the ND-FMT group (Figure 5F). Furthermore, the levels of total BAs, conjugated Bas, and unconjugated BAs in fecal excretion were significantly increased in the HFD-FMT group compared with the ND-FMT group, since unconjugated BAs, DCA, and LCA performed significantly increased levels, while conjugated BAs, TCA, and TDCA were significantly increased (Figure 5G). These results confirmed that gut microbiota contributed to BAs metabolic dysfunction during HFD-associated GDM development.

**Table 3:** FPG, FINS, and HOMA-IR were measured in mice from the ND-FMT and HFD-FMT groups.

	FPG (mmol/L)				FINS (μg/L)				HOMA-IR			
	D0	D6	D12	D18	D0	D6	D12	D18	D0	D6	D12	D18
ND-FMT	7.080 ± 0.888	7.300 ± 0.815	7.520 ± 0.592	10.070 ± 0.845	0.762 ± 0.108	0.866 ± 0.095	1.022 ± 0.158	1.074 ± 0.200	0.239 ± 0.038	0.281 ± 0.048	0.340 ± 0.048	0.481 ± 0.099
HFD-FMT	7.320 ± 0.863	8.570 ± 1.076	8.880 ± 0.649	12.310 ± 1.286	1.249 ± 0.170	1.287 ± 0.241	1.410 ± 0.363	1.243 ± 0.252	0.407 ± 0.077	0.488 ± 0.096	0.554 ± 0.142	0.683 ± 0.164
*t* value	0.5956	3.152	3.375	5.559	4.618	3.992	3.679	1.603	3.806	4.675	4.86	4.586
*p-*value	*p* > 0.05	*p* < 0.01	*p* < 0.01	*p* < 0.001	*p* < 0.001	*p* < 0.01	*p* < 0.01	*p* > 0.05	*p* < 0.01	*p* < 0.001	*p* < 0.001	*p* < 0.01

*p < 0.05 is significant.*

### HFD-associated gut microbiota disordered BAs metabolites impaired glycemia through *Fxr* pathway

**Figure 6:**
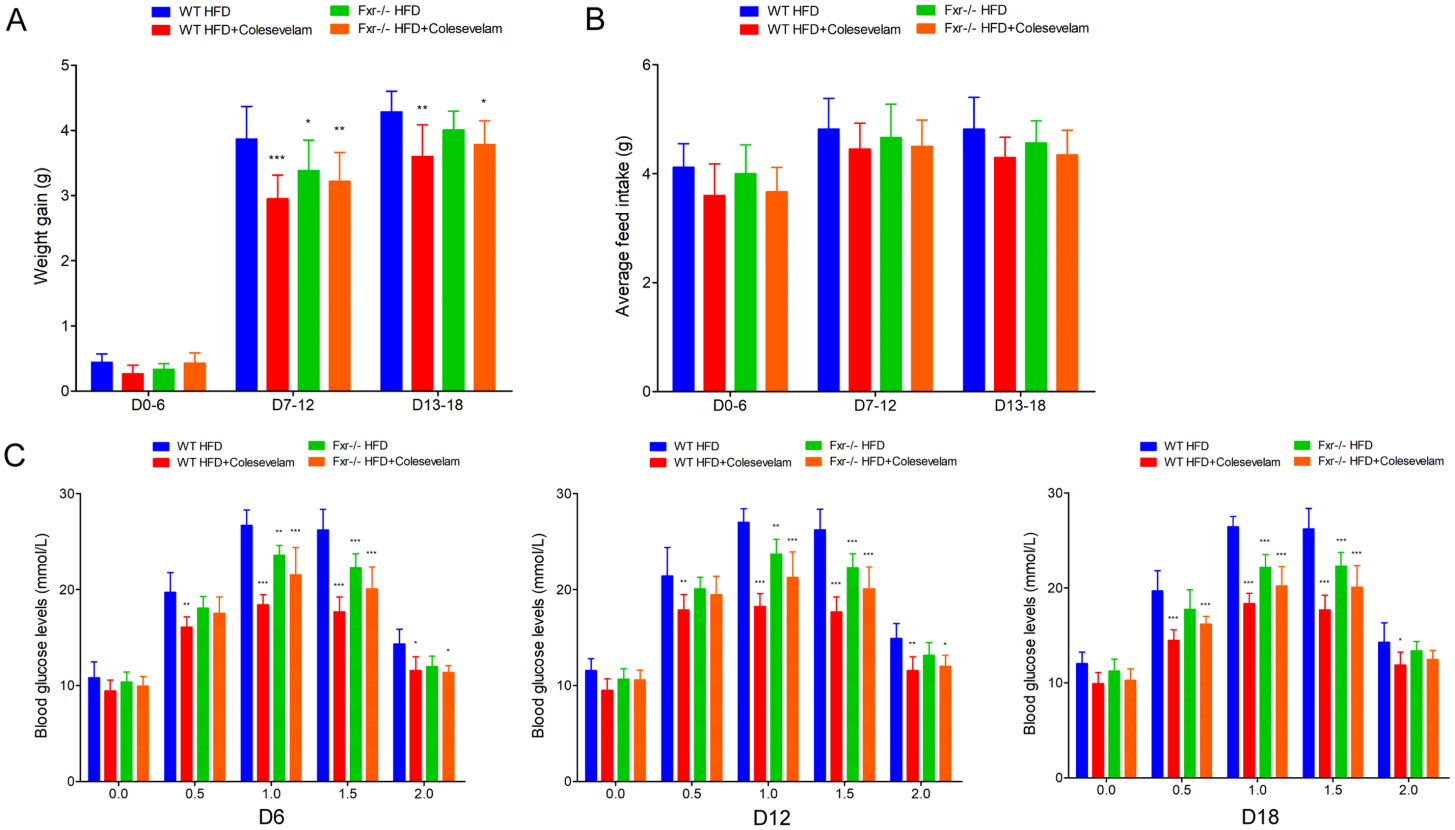
HFD-FMT administration disordered BAs metabolites impaired glycemia through Fxr pathway. **(A)** RT-qPCR was performed to examine mRNA expression levels of *Fxr*, *Shp*, and *Fgf15* in the terminal ileum, colon, liver, and serum of mice among each group. **(B)** Western blot showed protein expression levels of *Fxr*, *Shp*, and *Fgf15* in the terminal ileum, colon, and liver of mice among each group, and quantification was performed. **(C)** The line chart showed the body weight gain during different pregnancy periods among each group. **(D)** The barplot showed average feed intake during different pregnancy periods among each group. **(E)** The line chart showed the results of the OGTT during different pregnancy periods among each group. *p < 0.05; **p < 0.01; ***p < 0.001 means a significant difference versus the WT ND-FMT group.

It is well known that Fxr deficiency protected against diet-induced obesity and improved glucose homeostasis upon modulating BAs metabolites (Trabelsi et al., 2015; Prawitt et al., 2011). To test the response of HFD-associated gut microbiota disordered BAs metabolites through Fxr pathway, WT and *Fxr−/−* GDM mice were gavaged with stools from HFD-fed mice (HFD-FMT) or ND-fed mice (ND-FMT), along with periodic antibiotics cocktail treatment. As expected, HFD-FMT administration promoted Fxr activity in the terminal ileum, colon, and liver as reflected by the activation of *Shp* and *Fgf15* gene mRNA (Figure 6A) and protein expression (Figure 6B). Moreover, we found *Fgf15* was upregulated in the serum of mice from the WT HFD-FMT group than the mice from the WT ND-FMT group (Figure 6A). In addition, HFD-FMT administration significantly increased the body weight of WT mice in pregnancy (D7–D12 and D13–D18) (Figure 6C), average feed intake of mice during pregnancy (Figure 6D), disturbed the glucose metabolism (Figure 6E), and aggravated insulin resistance (Table 4), although the above effect was not observed in HFD-FMT *Fxr−/−* mice compared with ND-FMT *Fxr−/−* mice.

**Table 4:** FPG, FINS, and HOMA-IR were measured in mice among each group.

	FPG (mmol/L)				FINS (μg/L)				HOMA-IR			
	D0	D6	D12	D18	D0	D6	D12	D18	D0	D6	D12	D18
WT ND-FMT	8.617 ± 1.102	8.800 ± 1.002	9.500 ± 1.053	9.050 ± 0.718	1.023 ± 0.157	1.128 ± 0.238	1.023 ± 0.190	1.200 ± 0.222	0.391 ± 0.074	0.447 ± 0.130	0.439 ± 0.127	0.485 ± 0.108
WT HFD-FMT	9.867 ± 4.220	11.467 ± 1.417	12.100 ± 1.871	11.533 ± 1.337	1.463 ± 0.168	1.597 ± 0.214	1.488 ± 0.145	1.537 ± 0.105	0.655 ± 0.303	0.809 ± 0.120	0.800 ± 0.142	0.792 ± 0.140
Fxr−/− ND-FMT	8.567 ± 0.547	9.383 ± 1.211	9.300 ± 0.976	8.783 ± 0.808	0.813 ± 0.124	1.047 ± 0.191	0.865 ± 0.067	1.092 ± 0.134	0.311 ± 0.059	0.437 ± 0.099	0.358 ± 0.047	0.424 ± 0.048
Fxr−/− HFD-FMT	9.217 ± 0.578	10.833 ± 0.931	11.317 ± 1.789	10.883 ± 1.625	1.065 ± 0.144	1.187 ± 0.143	1.048 ± 0.096	1.210 ± 0.113	0.434 ± 0.045	0.571 ± 0.084	0.526 ± 0.086	0.590 ± 0.124
WT ND-FMT vs. WT HFD-FMT *t* value	1.319	2.813	2.743	2.62	4.41	4.694	4.661	3.374	3.462	4.762	4.754	4.04
WT ND-FMT vs. WT HFD-FMT *p-*value	*p* > 0.05	*p* < 0.05	*p* < 0.05	*p* < 0.05	*p* < 0.001	*p* < 0.001	*p* < 0.001	*p* < 0.01	*p* < 0.01	*p* < 0.001	*p* < 0.001	*p* < 0.001
Fxr−/− ND-FMT vs. Fxr−/− HFD-FMT *t* value	0.6857	1.53	2.128	2.215	2.522	1.403	1.838	1.186	1.625	1.756	2.216	2.182
Fxr−/− ND-FMT vs. Fxr−/− HFD-FMT *p-*value	*p* > 0.05	*p* > 0.05	*p* > 0.05	*p* > 0.05	*p* > 0.05	*p* > 0.05	*p* > 0.05	*p* > 0.05	*p* > 0.05	*p* > 0.05	*p* > 0.05	*p* > 0.05

*p < 0.05 is significant.*

### BAs sequestrant alleviated HFD-associated GDM development in mice

**Figure 7:**
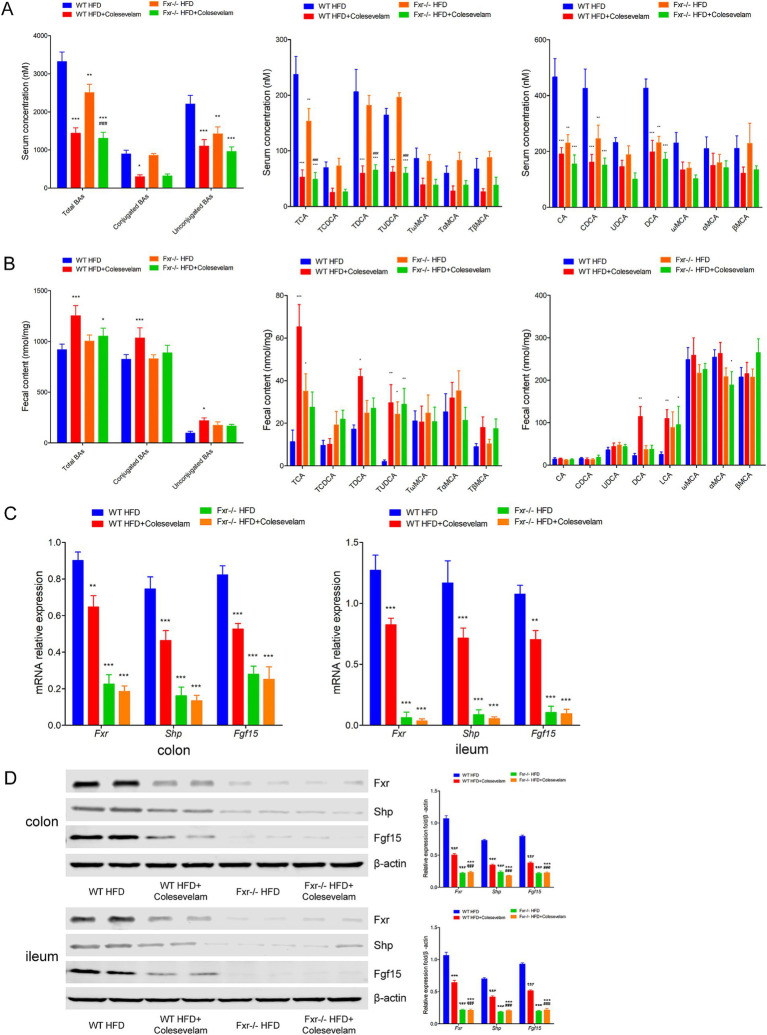
BAs sequestrant alleviated HFD-associated GDM development in mice. **(A)** The line chart showed the body weight gain during different pregnancy periods among each group. **(B)** The barplot showed average feed intake during different pregnancy periods among each group. **(C)** The line chart showed the results of the OGTT during different pregnancy periods among each group. *p < 0.05; **p < 0.01; ***p < 0.001 means a significant difference versus the WT HFD group.

To further explore gut microbiota associated BAs metabolic dysfunction promoting GDM development, we fed *WT* and *Fxr−/−* GDM mice by HFD, along with or without colesevelam intervention to enhance BAs metabolic. As shown in Figure 7A, colesevelam administration significantly decreased the body weight of *WT* mice in pregnancy fed with HFD (D7–D12 and D13–D18). However, colesevelam administration had no significant effect on average feed intake of mice during pregnancy (Figure 7B). To study the regulated effect of colesevelam on glucose metabolism during pregnancy, the OGTT test was performed on mice. Compared with the WT HFD group, the blood glucose levels of the mice in WT HFD + Colesevelam group on each time points of D6, D12, and D18 were significantly decreased (Figure 7C). Moreover, Table 5 depicts ameliorative insulin resistance throughout pregnancy in mice from the *WT* HFD + Colesevelam group compared to the WT HFD group. Interestingly, our data indicated colesevelam intervention performed improved effect on HFD-associated GDM development only in *WT* mice but not in the *Fxr−/−* HFD + Colesevelam group compared with *Fxr−/−* HFD group.

**Table 5:** FPG, FINS, and HOMA-IR were measured in WT and Fxr−/− GDM mice by HFD with or without colesevelam intervention.

	FPG (mmol/L)				FINS (μg/L)				HOMA-IR			
	D0	D6	D12	D18	D0	D6	D12	D18	D0	D6	D12	D18
WT HFD	11.333 ± 1.402	10.817 ± 1.656	11.550 ± 1.247	12.017 ± 1.219	1.502 ± 0.212	1.565 ± 0.099	1.665 ± 0.210	1.552 ± 0.097	0.763 ± 0.172	0.755 ± 0.139	0.857 ± 0.152	0.829 ± 0.102
WT HFD + Colesevelam	8.383 ± 0.668	9.433 ± 1.143	12.100 ± 1.871	11.533 ± 1.337	1.000 ± 0.130	1.132 ± 0.131	1.092 ± 0.160	1.200 ± 0.222	0.373 ± 0.057	0.474 ± 0.082	0.459 ± 0.072	0.526 ± 0.095
Fxr−/− HFD	9.567 ± 0.781	10.367 ± 1.052	10.667 ± 1.080	11.233 ± 1.268	1.313 ± 0.293	1.262 ± 0.169	1.492 ± 0.171	1.387 ± 0.082	0.558 ± 0.130	0.580 ± 0.085	0.706 ± 0.104	0.692 ± 0.081
Fxr−/− HFD + Colesevelam	9.133 ± 0.589	9.950 ± 0.997	10.583 ± 1.021	10.267 ± 1.206	1.065 ± 0.144	1.220 ± 0.127	1.320 ± 0.179	1.278 ± 0.158	0.434 ± 0.081	0.536 ± 0.049	0.619 ± 0.088	0.587 ± 0.121
WT HFD vs. WT HFD + Colesevelam *t* value	4.786	2.244	3.326	3.38	4.868	4.205	5.563	3.412	6.282	4.519	6.415	4.891
WT HFD vs. WT HFD + Colesevelam *p-*value	*p* < 0.001	*p* > 0.05	*p* < 0.01	*p* < 0.01	*p* < 0.001	*p* < 0.001	*p* < 0.001	*p* < 0.01	*p* < 0.001	*p* < 0.001	*p* < 0.001	*p* < 0.001
Fxr−/− HFD vs. Fxr−/− HFD + Colesevelam *t* value	0.7031	0.676	0.1352	1.568	2.419	0.4043	1.666	1.051	1.993	0.6996	1.416	1.685
Fxr−/− HFD vs. Fxr−/− HFD + Colesevelam *p-*value	*p* > 0.05	*p* > 0.05	*p* > 0.05	*p* > 0.05	*p* > 0.05	*p* > 0.05	*p* > 0.05	*p* > 0.05	*p* > 0.05	*p* > 0.05	*p* > 0.05	*p* > 0.05

*p < 0.05 is significant.*

### BAs sequestrant enhanced HFD-associated BAs metabolic through *Fxr* pathway in mice

**Figure 8:**
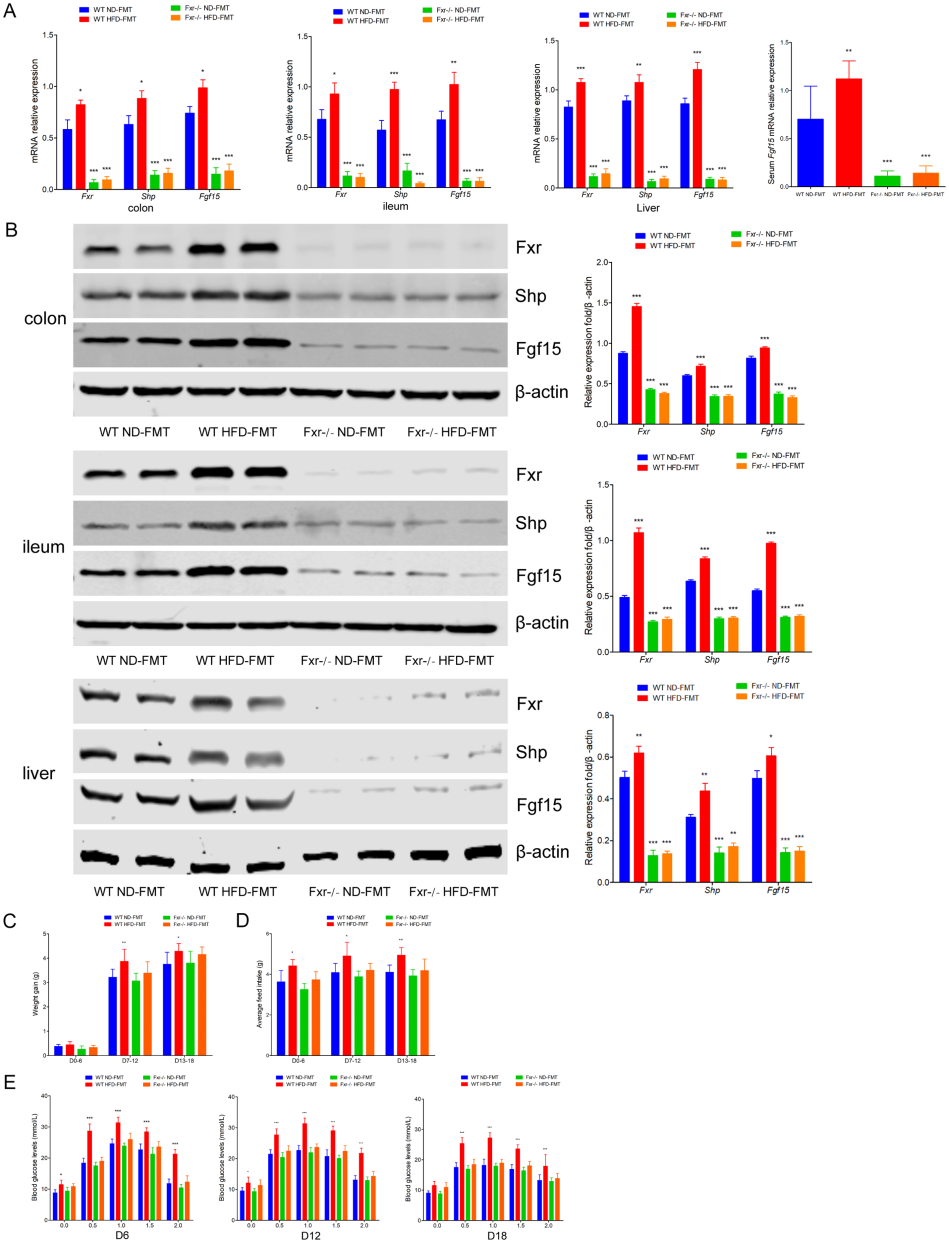
BAs sequestrant enhanced HFD-associated BAs metabolic through Fxr pathway in mice. **(A)** Serum BA classes and profile in mice among each group. **(B)** Fecal BA classes and profile in mice among each group. **(C)** Liver BA classes and profile in mice among each group. **(D)** RT-qPCR was performed to examine mRNA expression levels of *Fxr*, *Shp*, and *Fgf15* in the terminal ileum, colon, and liver of mice among each group. **(E)** Western blot showed protein expression levels of *Fxr*, *Shp*, and *Fgf15* in the terminal ileum, colon, and liver of mice among each group, and quantification was performed. *p < 0.05; **p < 0.01; ***p < 0.001 means a significant difference versus the WT HFD group.

To comprehensively understand the mechanism through which BAs sequestration protected HFD-associated GDM development, we fed *WT* and *Fxr−/−* GDM mice by HFD, along with or without colesevelam intervention. We found the levels of total BAs, conjugated Bas, and unconjugated BAs in serum of *WT* mice were dramatically decreased after colesevelam treatment, specifically performed as increased levels of CA, CDCA, and DCA, along with conjugated BAs, TCA, TDCA, and TUDCA in mice fed with HFD (Figure 8A). As shown in Figure 8B, BAs sequestration treatment significantly increased total BAs, conjugated Bas, and unconjugated BAs in fecal excretion from HFD-fed *WT* mice, indicating that colesevelam indeed interfered with intestinal BA absorption in mice as shown by unconjugated BAs, UDCA, DCA, ωMCA, and LCA performed significantly increased levels, while conjugated BAs, TCA, and TDCA were significantly increased. In addition, as shown in Figure 8C, BAs sequestration treatment significantly decreased total BAs and conjugated BAs in liver from WT mice fed with HFD, indicating that colesevelam promoted BAs metabolic in the liver of mice as shown by unconjugated BAs, CA, DCA, LCA, and αMCA, performed significantly decreased levels, while conjugated BAs, TCA, and TDCA were significantly decreased. Then, we analyzed the mRNA and protein expression levels of *Fxr*, *Shp,* and *Fgf15* in the terminal ileum, colon, and liver of mice. *Fxr* expression was decreased in the *WT* HFD + Colesevelam group compared to mice in the *WT* HFD group. In line with the effective prevention of intestinal BAs uptake, the expression of the *Fxr* downstream target genes *Shp* and *Fgf15* was strongly reduced upon colesevelam treatment in the WT HFD + Colesevelam group compared to mice in the WT HFD group (Figures 8D,E). Moreover, we found *Fgf15* was downregulated in the serum of mice from the *WT* HFD + Colesevelam group than the mice from the *WT* HFD group (Figure 8D). Notably, the above promoted effect of colesevelam intervention on HFD-associated BAs metabolic was not obviously changed in the *Fxr−/−* HFD + Colesevelam group compared with the *Fxr−/−* HFD group.

### BAs metabolic involved in mediating HFD-associated GDM development through Fxr pathway in mice

**Figure 9:**
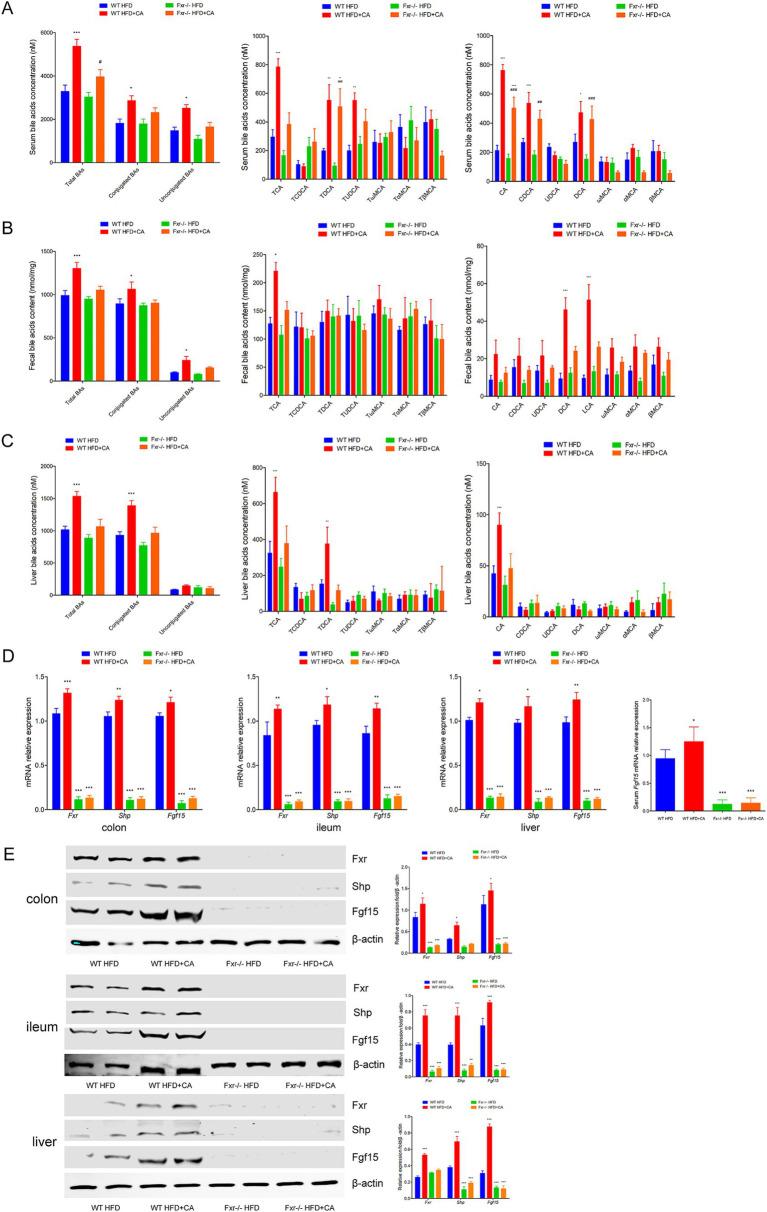
BAs metabolic involved in mediating HFD-associated GDM development through Fxr pathway in mice. **(A)** Serum BA classes and profile in mice among each group. **(B)** Fecal BA classes and profile in mice among each group. **(C)** Liver BA classes and profile in mice among each group. **(D)** RT-qPCR was performed to examine mRNA expression levels of *Fxr*, *Shp*, and *Fgf15* in the terminal ileum, colon, and liver of mice among each group. **(E)** Western blot showed protein expression levels of *Fxr*, *Shp*, and *Fgf15* in the terminal ileum, colon, and liver of mice among each group, and quantification was performed. *p < 0.05; **p < 0.01; ***p < 0.001 means a significant difference versus the WT HFD group.

To further illuminated BAs metabolic was involved in regulating HFD-associated GDM development, we fed *WT* and *Fxr−/−* GDM mice by HFD, along with or without BAs intervention. We found the levels of total BAs, conjugated Bas, and unconjugated BAs in serum of *WT* mice were dramatically increased after CA treatment, specifically performed as increased levels of CA, CDCA, and DCA, along with conjugated BAs, TCA, TDCA, and TUDCA in mice fed with HFD (Figure 9A). As shown in Figure 9B, CA treatment significantly increased total BAs, conjugated Bas, and unconjugated BAs in fecal excretion from HFD-fed *WT* mice, as shown by unconjugated BAs, DCA, and LCA, performed significantly increased levels, while conjugated BAs and TCA was significantly increased. In addition, as shown in Figure 9C, CA treatment significantly increased total BAs and conjugated BAs in liver of WT mice fed with HFD, as shown by unconjugated BAs, CA performed significantly increased levels, while conjugated BAs, TCA, and TDCA were significantly increased. Then, we analyzed the mRNA and protein expression levels of *Fxr*, *Shp,* and *Fgf15* in the terminal ileum, colon, and liver of mice. *Fxr, Shp,* and *Fgf15* were strongly increased upon CA treatment in the WT HFD + CA group compared to mice in the *WT* HFD group (Figures 9D,E). Furthermore, the expression level of *Fgf15* was significantly increased in the serum of mice from the *WT* HFD + CA group than the mice from the *WT* HFD group (Figure 9D). Interestingly, CA intervention failed to perform the above effect on HFD-associated BAs metabolic and the expression of *Fxr, Shp,* and *Fgf15* in the *Fxr−/−* HFD-fed mice.

## Discussion

It was well established that western diet was associated with high risk of GDM (Mizgier et al., 2021; Mijatovic-Vukas et al., 2018). Epidemiological data also indicated that HFD often leads to obesity and increased risk of GDM (Cole et al., 2021b). However, the mechanism of HFD inducing GDM development was largely unknown. In the current study, we initially confirmed that HFD promoted GDM and predicated high prevalence of complication clinically. Since accumulated literature indicated that excess dietary fat intake induced imbalanced gut microbiome, which was associated with angiocardiopathy, diabetes mellitus, and cancers (Hemmati et al., 2023; Paone et al., 2022). We found gut microbiota depletion by antibiotics neutralized the crippled effect of excess fat intake on glucose metabolism in HFD-fed GDM mice and further confirmed the direct effect of HFD-modulated gut microbiota contributes to GDM development by FMT in GDM mice along with periodic antibiotics treatment to execute bacteria depletion. In the current study, we found *Alistipes onderdonkii* was one of the most obviously enriched while *Akkermansiaceae* was one of the most significantly reduction in the GDM patients with HFD. Moreover, the abundance of potential pathogenic bacterial species *Alistipes* sp. was also one of the most significantly higher in mice fed with HFD and *Akkermansiaceae* was one of the most significantly depleted in HFD-fed mice. Importantly, we further confirmed that *Alistipes* sp. was also one of the most significantly higher and *Akkermansiaceae* was one of the most significantly depleted in HFD-FMT mice. Genera of *Alistipes* were mostly associated with mammalian habits and were already reported to be altered in cancer, irritable bowel syndrome, animal-based diet, and high-fat consumption (Yang et al., 2022; Daniel et al., 2014; Saulnier et al., 2011). Statistical and computational analyses of the metagenomic data identified four potential bacterial markers associated with GDM, being *Alistipes putredinis* among them (Fenner et al., 2007). The reduced abundance of *Alistipes* also appeared in type 2 diabetes mellitus (T2DM), reinforced its modulation effect on metabolomics in intestine (Wu et al., 2020). Meanwhile, subsequent research conducted on humans and rodents all converged to indicate a recurrent under-representation of *Akkermansiaceae muciniphila* in the gut of individuals with obesity when compared with lean counterparts (Fernandez-Quintela et al., 2023; Crovesy et al., 2020). Metabolic disorders, including obesity and T2DM, have been associated with a reduction in the abundance of *Akkermansiaceae muciniphila* in rodent and human studies (Macchione et al., 2019; Santacruz et al., 2010). It was demonstrated that daily administration of live *Akkermansiaceae muciniphila* in mice alleviated HFD-induced metabolic disorders and insulin resistance (Li et al., 2017). In addition, altered gut microbiota composition during pregnancy acted as a characteristic of GDM (Everard et al., 2013). In most women, altered in gut microbiota composition comprised a rise in *Proteobacteria* (Ferrocino et al., 2018). The abundance of *Firmicutes* and *Bacteroidetes* was scarce among GDM women (Koren et al., 2012), although Wang et al. have noted that there was an increase of *Firmicutes* and *Bacteroidetes* ratio in GDM women (Wang et al., 2024).

It was reported that diets high in carbohydrate, particularly fat, stimulated BAs secretion (Wang et al., 2018). Others also reported HFD-induced gut microbial changes and increased secondary BAs in a mouse model of hepatocellular carcinoma and colon cancers (Meyer et al., 1976). Established viewpoints reminded that perturbations in the gut microbiome could affect the BAs pool size and composition, which were linked to metabolic disorders (Li et al., 2022) and the development of metabolism-related gastrointestinal inflammation, fatty liver disease, even cancers (Collins et al., 2023; Jia et al., 2018; Ma et al., 2018). It has been reported that serum levels of DCA were increased by an HFD and reduced by antibiotic treatment, which provided a functional link between the gut microbiota and luminal BAs in mice (Wu et al., 2021). A study about obesity susceptibility found that *Clostridium scindens* was positively correlated with LCA, DCA, and UDCA (Ushiroda et al., 2019). Bacteria with bile salt hydrolase activity, including *Bacteroides, Clostridium, Lactobacillus, Bifidobacterium, Enterococcus, Ruminococcaceae,* and *Listeria*, could dominate the bile acid deconjugation process (Wei et al., 2020; Ridlon et al., 2006; Guzior and Quinn, 2021). Furthermore, *Clostridium* and *Eubacterium* were found to contribute to bile acid dehydroxylation; *Bacteroides*, *Eubacterium*, *Clostridium*, *Escherichia*, *Eggerthella*, *Eubacterium*, *Peptostreptococcus,* and *Ruminococcus* were confirmed to be associated with their oxidation and isomerization (Collins et al., 2023), while *Bacteroides*, *Eubacterium,* and *Lactobacillus* contributed to their esterification, and *Clostridium*, *Fusobacterium*, *Peptococcus,* and *Pseudomonas* contributed to their desulfation (Thomas et al., 2022). In turn, BAs effectively regulated the growth of the gut microbiota and maintained intestinal homeostasis (Long et al., 2017). Except for damaging on bacterial membranes, BAs could also indirectly inhibit microbial growth by regulating the expression of nitric oxide synthase and antimicrobial peptide genes (Li et al., 2021). Thus, our present study indicated that HFD disturbed BAs metabolic disorders contributed to GDM development at least partly induced by gut microbial dysbiosis, although exactly bacterial genus should be further investigated to facilitate GDM development.

In addition to the ability to directly convert primary bile acids to secondary bile acids, the gut microbiota could also control bile acid synthesis enzymes. BAs were endogenous signaling molecules which binded to the BA receptors, Fxr, to regulate BA homeostasis in the enterohepatic circulation and maintain glucose homeostasis (Ridlon et al., 2016). Unconjugated BAs such as CA, DCA, and UDCA were the mainly potent endogenous ligands of Fxr (Houten et al., 2006). The gut microbiota and BAs metabolism were closely interrelated and directly modulated by the intestinal-hepatic Fxr-Fgf15 signaling axis. T-βMCA and T-αMCA were identified as naturally occurring Fxr antagonists, and reduced levels of T-βMCA led to a decreased production of Fgf15 (Claudel et al., 2005). Antibiotics-induced decrease of ileal Fxr signaling was recovered by administration of unconjugated CA, indicating the importance of BA deconjugation for Fxr activation (Sayin et al., 2013). Recently, BA sequestrant treatment has been shown to alleviate blood glucose in T2DM patients (Kuribayashi et al., 2012). An increasing body of evidence indicated that BA pool size and composition controlled glucose homeostasis through BA receptors. It was reported that downregulation of proglucagon in intestines was the result of Fxr activation in L-cells rather than an indirect effect on bile acid pool modification. Moreover, Fxr activation could inhibit the activation of proglucagon induced by food intake (Fonseca et al., 2010). Fxr activation in the intestine promoted *Fgf15/19* secretion which, in addition to its role in the regulation of BA synthesis, reduced adiposity and improved brown adipose tissue energy expenditure and the metabolic rate in mice (Daoudi et al., 2011). It was also reported that the increased expression of intestinal proglucagon upon Fxr deficiency was depending on the gut microbiota (Tomlinson et al., 2002). Our current data indicated inactivation of Fxr protected against HFD-FMT administration induced GDM development and had an improved effect on glucose metabolism as reflected by the above reaction only performed in WT mice but not in whole-body Fxr-deficient mice, which suggested that Fxr pathway was involved in the response of HFD-associated gut microbiota disordered BAs metabolites induced GDM development.

Colesevelam, being a BAs sequestrant, binded BAs in the intestine, lead to the formation of non-absorbable complexes that were excreted into the feces thereby promoted fecal BA excretion (Trabelsi et al., 2015). It was reported the increasing of TDCA in livers of *Cyp2c70−*/*−* mice upon pharmacological inhibition of the ileal BA transporter performed an enhanced effect on BA hydrophobicity despite increased 12α−/non-12α-hydroxylated BA ratios (Stroeve et al., 2010). Moreover, Palmiotti et al. found colesevelam treatment resulted in a significantly decreased hydrophobicity of biliary BAs (Truong et al., 2022). Furthermore, previous study shown that colesevelam treatment increased the metabolic clearance rate of glucose in diabetic *db*/*db* mice by improving insulin sensitivity (Palmiotti et al., 2023). Our present study confirmed colesevelam treatment alleviated HFD-associated GDM development through improving BAs metabolic at least partly via Fxr pathway in mice. In addition, we further confirmed that BAs metabolic involved in mediating HFD-associated GDM development through fed *Fxr−/−* GDM mice by HFD along with BAs intervention. Moreover, our data demonstrated a parallel trend of blood glucose and plasma insulin levels during pregnancy, which provided the evidence of insulin resistance was the main occurrence mechanism in HFD-associated GDM. In contrast to our current observations, Palmiotti et al. found colesevelam treatment had no effects on plasma insulin levels either on glucose excursions during an OGTT in mice (Meissner et al., 2011). We hypothesized that colesevelam treatment could perform different effects on glucose homeostasis and insulin sensitivity in GDM mice fed with HFD. Moreover, it was unclear whether HFD-associated gut microbiota alterations disordered the composition of BA profile and interfered the activation of Fxr pathway in the liver or pancreas of GDM mice, which will be further investigated in the future.

Although our data indicated instrumental delivery, perineal tears, postpartum hemorrhage, difficulty initiating, and maintaining breastfeeding had no association with HFD, however, that could not rule out HFD was the risk of these complications. We thought this negative result was restricted by the amount of samples. Actually, germ-free mice, which was depleted of their resident microbiota, was considered as the gold standard for exploring the role of the microbiome in health and disease. However, germ-free mice got limited value in the study of human-specific pathogens because they do not support their replication (Wahl et al., 2023). In our studies, we just provided some evidence of gut microbiota alteration, including but not limited to enrichment of *Alistipes onderdonkii*, *Lachnospiraceae bacterium 1_7_58FAA*, *Clostridium aspaaragiforme*, reduction of *Akkermansiaceae, Paraprevotell xylaniphila, and Prevotella copri* in GDM patients, along with enrichment of *Alistipes* sp. *Marseille-P5997, Bacteroidetes uniforms, Bacteroidetes* sp. *A1C1*, depletion of *Lachnospiraceae, Akkermansiaceae, Lactobacillaceae* in GDM mice, participated in contributing HFD-induced GDM development. Further study will be focused on the effect of each gut microbiota during GDM progression.

In summary, our study confirmed that HFD induced GDM and contributed to poor prognosis in GDM patients through inducing gut microbial dysbiosis and metabolic alteration. Among these metabolic alterations, BAs profile altered was involved in modulating HFD inducing GDM development regulated by gut microbiota. Moreover, Fxr pathway participated in regulating HFD-associated gut microbiota disordered BAs metabolites and aggravating GDM in mice. Modulating gut microbiota and BAs metabolites could be a potential therapeutic strategy in the prevention and treatment of HFD-associated GDM.

## Data Availability

The datasets presented in this study can be found in online repositories. The names of the repository/repositories and accession number(s) can be found in the article/Supplementary material.

## Footnotes

^1^IDQC, www.yyjy365.org/diet.

^2^ www.metacpan.org
