# Supplementary material for: High-fat diet promotes gestational diabetes mellitus through modulating gut microbiota and bile acid metabolism
Source: Front Microbiol. 2025 Jan 28;15:1480446. doi: 10.3389/fmicb.2024.1480446 (PMC11810896; doi:10.3389/fmicb.2024.1480446)
Supplement: Supplementary file 1 [file Table_1.DOC]

Supplemental Table 1: Primers used for RT-qPCR

| **Primer Name** | **Primer Sequence: 5'-3'** |
| --- | --- |
| mice Forward Fxr | GCACGCTGATCAGACAGCTA |
| mice Reverse Fxr | CAGGAGGGTCTGTTGGTCTG |
| mice Forward Shp | GTACCTGAAGGGCACGATCC |
| mice Reverse Shp | GTGAAGTCTTGGAGCCCTGGT |
| mice Forward Fgf 15 | CAGTCTTCCTCCGAGTAGCG |
| mice Reverse Fgf15 | TGAAGACGATTGCCATCAAG |
| Forward β-actin | ATGTTGAGACCTTCAACACC |
| Reverse β-actin | AGGTAGTCAGTCAGGTCCCGGCC |
